# Multimodal rehabilitation for hard flaccid syndrome in a retrospective case series

**DOI:** 10.3389/fresc.2026.1970062

**Published:** 2026-09-29

**Authors:** Christopher L. Mendias, Tariq M. Awan

**Affiliations:** Performance Medicine Institute, Phoenix, AZ, United States

**Keywords:** chronic pelvic pain disorder, erectile dysfunction, hard flaccid syndrome, male sexual dysfunction, pelvic floor rehabilitation, sexual rehabilitation

## Abstract

**Introduction:**

Hard flaccid syndrome (HFS) is an emerging condition of male sexual dysfunction characterized by a persistent semi-rigid penis in the flaccid state, altered penile sensation, erectile dysfunction, and pelvic or perineal pain. Single-modality treatments have shown limited success, and multimodal protocols have been reported only in single-patient case studies.

**Aim:**

To describe clinical outcomes associated with completion of an integrative multimodal rehabilitation protocol in a retrospective case series of men with HFS.

**Methods:**

Thirty-two men with HFS completed a comprehensive protocol combining class IV laser therapy, dry needling, radial pressure wave shockwave therapy, therapeutic ultrasound, biofeedback training, manual therapy, therapeutic exercise, behavioral coaching, and daily oral tadalafil. Patient-reported outcomes were collected at treatment initiation and completion.

**Outcomes:**

Erection Hardness Scale (EHS), penile function satisfaction, PROMIS Interest in Sexual Activity, and PROMIS Global Health Physical and Mental Component scores.

**Results:**

Median EHS increased from 2 to 4 and median penile satisfaction increased from 2 to 5 (both *P* < 0.01). All 32 patients achieved EHS ≥3 by treatment end, compared to 8 of 32 (25%) at baseline. PROMIS Interest in Sexual Activity, Global Physical Health, and Global Mental Health scores all improved significantly (*P* < 0.01). Common comorbid features included low back pain (53%), hip or groin pain (38%), pelvic floor pain (31%), and urinary symptoms (28%).

**Discussion:**

In this retrospective case series, completion of a multimodal rehabilitation program was associated with improvements in erection hardness, penile satisfaction, sexual interest, and global health in men with HFS. These hypothesis-generating findings warrant prospective controlled studies evaluating efficacy, durability, and component-specific effects.

## 1 Introduction

Hard flaccid syndrome (HFS) is an emerging condition of male sexual dysfunction characterized by a persistent semi-rigid quality of the flaccid penis, altered penile sensation, erectile dysfunction, and pelvic pain (1, 2). Population incidence and prevalence estimates are unavailable because standardized diagnostic criteria and studies at the population level have not been established. HFS can impair sexual function, including ejaculation, morning erections, libido, urinary symptoms, and partner relationships (1–4). HFS is proposed to fit within the spectrum of persistent genital arousal disorder and genito-pelvic dysesthesia (PGAD/GPD) syndromes (5) in which symptoms may arise from end organ, pelvic and perineal, cauda equina, spinal cord, or brain regions that converge on the pelvic-pudendal-hypogastric reflex (5). Symptom-directed monotherapies, including nerve blocks, muscle relaxants, and pain medication, have generally shown limited success (2), while limited case reports suggest that multimodal rehabilitation, pain neurophysiology education, and graded exposure may improve symptoms (6–8). Whether this approach produces consistent symptom resolution across a broader HFS population remains unknown.

Multimodal treatments integrating physical, neurophysiological, and behavioral mechanisms have shown benefit in other penile disorders, as well as chronic pelvic pain and musculoskeletal conditions (9–11). We therefore designed a treatment protocol anchored in neurophysiology, biomechanics, exercise physiology, connective tissue mechanobiology, and behavioral therapy. We report outcomes from 32 men with HFS treated with this protocol and propose a hypothesis-generating refinement of the PGAD/GPD framework (5) that accounts for the convergent phenotype, heterogeneous contributing factors, and consistent direction of treatment response observed in this cohort.

## 2 Materials and methods

### 2.1 Participants and procedures

This retrospective case series included 32 men treated at our clinic for hard flaccid syndrome. The study adhered to the Declaration of Helsinki. This retrospective case series was reviewed by Pearl IRB (Fishers, IN) and determined to be exempt under 45 CFR 46.104(d) (4), Secondary Research Uses of Data or Specimens (IRB Protocol #2026-0350). Patients were eligible if they reported a persistent or recurrent semi-rigid quality of the flaccid penis on most days for at least 3 months, with one or more of the following: altered penile sensation, erectile dysfunction, ejaculatory dysfunction, or perineal or pelvic pain. Patients were excluded if symptoms were better explained by acute penile fracture or surgically urgent trauma, active genitourinary infection, urethritis, prostatitis, epididymitis, testicular torsion, malignancy, clinically dominant Peyronie's disease, temporally related pelvic or penile surgery, neurologic disease known to affect pelvic or sexual function, or uncontrolled endocrine, cardiovascular, or psychiatric disease likely to independently explain sexual dysfunction. Patients were also excluded for incomplete baseline or follow-up outcomes, inability to participate in treatment, or insufficient documentation to confirm diagnosis or treatment course.

### 2.2 Qualitative content of narratives of history of hard flaccid symptoms

Self-reported histories were analyzed using conventional inductive qualitative content analysis (12). Narratives from the electronic medical record were reviewed in full, meaningful text segments were coded, and codes were iteratively organized into 23 factors and 4 themes. Each history was then scored for the presence or absence of each factor, and theme prevalence was summarized descriptively. Patients also reported prior HFS treatments and rated perceived change in symptoms using the 7-point Patient Global Impression of Change (PGIC) scale (13). Modalities used by fewer than 3 patients were excluded from the prior treatment analysis.

### 2.3 Examination

A focused physical examination was performed at the initial visit. Genital examination assessed penile shape, contour, temperature, color, and the presence of semi-rigidity in the flaccid state. Testicular and scrotal examination assessed size, position, tenderness, varicocele, hydrocele, epididymal abnormalities, and tenderness along the spermatic cord and inguinal canal. Lumbosacral and hip examination included manual muscle testing, special tests, and provocative maneuvers, with radiographs or magnetic resonance imaging obtained when clinically indicated. External pelvic floor examination assessed muscle and fascial tone and tenderness. Sensory testing was performed over the penis and lower abdomen using a 10 g monofilament to assess A*β* fiber-mediated light touch and pressure sensation, a 128 Hz tuning fork to assess A*β* fiber-mediated vibratory sensation, and a Tip Therm device (US Neurologicals, Poulsbo, WA) for warm-cold discrimination to assess A*δ* and C fiber-mediated thermal discrimination (14, 15). Sensory testing was also performed on dermatomes of patients with suspected pathology of a particular spinal level. Cremasteric, bulbospongiosus, anal wink, and lower extremity deep tendon reflexes were also assessed.

### 2.4 Education

Patients received handouts and provider-led education on penile anatomy, erection and ejaculation physiology, current HFS knowledge, recovery after more severe penile injuries, pain neurophysiology, and psychosocial factors in HFS (16–18). The goals were to improve understanding, support shared decision-making, build confidence in self-management, reduce fear and maladaptive beliefs, and improve treatment adherence (19).

### 2.5 Counseling for self-reported high frequency, prolonged, or high force masturbation

Because some patients with HFS report high frequency, prolonged, or high force masturbation that may aggravate symptoms through repetitive mechanical loading (2, 4), interested patients received counseling focused on healthy, self-directed goals (20). This counseling reviewed the applied neurobiology of compulsive sexual behaviors (21), population ranges for masturbation frequency and duration (22), and individualized step-down plans over 4 to 8 weeks. Patients reporting problematic pornography use received education on sexual cue reactivity (21), population ranges for pornography use (23), and self-directed reduction strategies (24).

### 2.6 Sexual activity modification, return to sexual activity, and dating

Because HFS can lead to avoidance of sexual activity, partner withdrawal, and dating hesitancy (1, 2), structured coaching supported graded return to sexual activity. Goals included reducing avoidance-driven deconditioning, limiting catastrophizing, supporting relationship health, and restoring satisfying sexual function. Patients in relationships were counseled to temporarily reduce symptom-provoking mechanical loading by modifying frequency, duration, and intensity of intercourse or masturbation, while maintaining non-coital intimacy when appropriate (25). Single patients received coaching on dating hesitancy, normalization of performance anxiety after dysfunction, graded exposure to dating situations, partner communication, expectation setting, and management of performance-related anxiety (26). Both partnered and single patients received guidance on partner communication strategies, including how to disclose the condition to a partner when appropriate, how to set expectations during the recovery period, and how to manage performance-related anxiety during initial return to sexual activity (27).

### 2.7 Patient-reported outcome measures

Patient-reported outcomes were collected at treatment initiation and completion. PROMIS Global Health 10 was used to assess general physical and mental health, with physical component summary (PCS) and mental component summary (MCS) scores calculated from the overall assessment (28). PROMIS Brief Profile Male Interest in Sexual Activity short form was used to quantify sexual desire and complement erectile function outcomes by capturing psychological and motivational dimensions of sexual dysfunction in HFS (29). Raw scores were converted to T-scores for analysis. Erectile function was assessed using the Erection Hardness Scale (EHS), an ordinal scale ranging from 0 to 4 (30). Overall penile function satisfaction was assessed using a 5-point Likert scale (31, 32) in response to the question, “How satisfied are you with the overall function of your penis?” Response options were: 1, very dissatisfied; 2, dissatisfied; 3, neutral; 4, satisfied; and 5, very satisfied.

### 2.8 Treatment interventions

Patients underwent treatment sessions of seven synergistic modalities at each treatment session: (i) class IV laser therapy, (ii) radial pressure wave shockwave therapy, (iii) therapeutic ultrasound, (iv) dry needling, (v) manual therapy, (vi) biofeedback training, and (vii) therapeutic exercises. Oral tadalafil 5 mg daily was also prescribed to support NO/cGMP-mediated smooth muscle and myofibroblast relaxation and vascular function (33). Treatment sessions occurred one to two times per week. The protocol used the same core treatment categories across patients, while anatomic targets, dosing outside the penile region, and adjunctive techniques were individualized according to symptoms, examination findings, and tolerance. Patients were generally encouraged to continue treatment until they reached a satisfied or very satisfied state with overall penile function.

Class IV laser therapy (REMY Medical Laser, Cherry Hill, NJ) was applied to the penile shaft, targeting the corporal bodies, as a photobiomodulation intervention intended to support nitric oxide-mediated microvascular signaling and modulate neuromuscular or autonomic contributors to tissue tightness (34–37). In patients with perineal, scrotal, hip, groin, or low back symptoms, treatment was also applied to those areas. Penile treatment delivered approximately 700 to 800 J, distributed approximately evenly between 810 nm and 980 nm wavelengths. Dosing for other regions was adjusted to tissue size and symptoms.

Radial pressure wave shockwave therapy (Intelect RPW 2, Enovis, Wilmington, DE) was delivered to the dorsal and lateral aspects of the penis. A concave RO40 transmitter head (Enovis) was used to target energy to the superficial corpora cavernosa. Radial pressure wave therapy was selected because its divergent, superficial field can distribute mechanical stimulation across a broader region of myofascial tightness than the concentrated, depth-targeted field of focused shockwaves (38, 39). The frequency was 4–6 Hz which was selected based on studies that demonstrated effective myofascial spasticity reduction at this frequency (40). Each session delivered 1,000 to 2,000 pulses, with intensity adjusted to just below the maximum tolerated comfort level.

Therapeutic ultrasound was performed to improve blood flow and reduce fascial tightness, modified from studies which used this modality for the treatment of Peyronie's disease (41). Ultrasound (CX2, Richmar, Clayton, MO) was delivered at a frequency of 3Mhz with an energy output of 1.0–1.2 W/cm^2^ with a 100% duty cycle for up to 8 min.

Superficial dry needling was used as a low-irritability neuromodulatory intervention intended to reduce sympathetic guarding, improve autonomic tone, and decrease superficial fascial sensitivity and tightness (37, 42–44). Needles (SERIN, Boston, MA) were sterile, only used once, and occasional pistoning was performed as needed. To target penile fascia, needles (SERIN, Boston, MA) were placed in an evenly distributed pattern on the dorsal and lateral aspects of the penis to abut but not penetrate dartos fascia. For patients reporting perineal symptoms, dry needling of the pelvic floor was performed (45). Gentle heat, provided from infrared lamps, was applied during needling sessions. Dry needling was also performed over the lower abdominal area superficial to the ilioinguinal nerve, genital branch of the genitofemoral nerve, and the iliohypogastric nerve, which provide sensory innervation to the genitalia, as well as the hip and low back in patients who were symptomatic in these areas.

Manual therapy and proprioceptive neuromuscular facilitation (PNF) stretching were used to address myofascial and neuromuscular contributors to pelvic floor and perineal tension (46, 47). Manual therapy techniques included myofascial release and instrument-assisted soft tissue mobilization of the pelvic floor and lower abdominal muscles. For patients with comorbid lumbosacral or hip pathologies, joint mobilizations were performed when clinically appropriate. Patients were instructed on how to perform gentle transverse soft tissue mobilization on penile fascia at home. PNF stretching of the adductors, abductors, flexors, extensors, and internal rotators of the hip joint was performed.

Biofeedback training was used to reduce pelvic floor tension by providing real-time feedback on muscle activity, improving awareness, voluntary control, and coordination between contraction and relaxation (48). Training lasted up to 10 min and emphasized relaxation or downtraining using a noninvasive device (Boost, kGoal, Portland, OR). Patients with extensive perineal tension, or who developed tension after ejaculation, were instructed on applying a low frequency mechanical oscillating device (We-Vibe Tango X, Lovehoney Group, Atlanta, GA) parallel to the pelvic floor muscles.

Therapeutic exercise was conducted over three progressive stages, with patients spending 2–6 weeks at each stage, based on symptom resolution and functional capacity (Table 1). Exercises were selected to improve strength and stability, as well as activating somato-autonomic reflex pathways at spinal segments where low-threshold muscle afferents can produce segmental sympathoinhibition of penile vascular tone, enhance parasympathetic pro-erectile outflow, and drive activity-dependent neuroplastic reductions in sympathetic dominance (7, 37, 49–51). Additional exercises were selected to treat patients with lumbosacral (52) or hip (53, 54) musculoskeletal pathologies. In patients with neuromuscular inhibition, a Neubie direct current stimulator (NeuFit, Austin, TX) (55) was used to facilitate muscle contractions. Patients were instructed to perform prescribed exercises and stretching at home 2–3 times weekly.

**Table 1:** Therapeutic exercises. Therapeutic exercises used in this study across three stages of progression.

Stage	Hard Flaccid Syndrome Only	Additional Exercises for Hip Pathology	Additional Exercises for Low Back Pathology
Stage 1 - Relaxation and low-load control	90/90 bridge with ball and balloon, cat-cow, diaphragmatic breathing with pelvic-floor relaxation, glute bridge, heel slide, open-book thoracic rotation, pelvic tilts, quadruped rocking, supine march	Clamshell, isometric adduction, quadruped hip extension, side-lying hip abduction, standing hip abduction	Bent-knee fallout, bird dog, dead bug, hook-lying lower trunk rotation
Stage 2 - Stabilization and controlled loading	Bird dog, dead bug, modified front plank, Pallof press, reverse lunge, side plank, single-leg bridge, squat, step-up	Hip hike, lateral band walk, monster walk, short-lever Copenhagen plank, single-leg balance, step-down	Body blade stabilization, modified curl-up, quadruped row, stability-ball bridge, Swiss-ball rollout
Stage 3 - Integrated strength and return to activity	Bulgarian split squat, cable chop, farmer carry, medicine-ball slam, Pallof press with step-out, Romanian deadlift, sled push, Swiss-ball rollout	Copenhagen plank, lateral lunge, lateral or multiplanar stepping, loaded reverse lunge, loaded squat, loaded step-down, single-leg Romanian deadlift, single-leg squat to box	Back extension, bicycle crunches, body blade rotations, double-leg raise, Russian twists, single-arm cable row, suitcase carry

### 2.9 Statistical analyses

Because EHS and penile function satisfaction were ordinal outcomes and normality could not be assumed for the PROMIS outcomes in this cohort, we used two-tailed Wilcoxon matched-pairs signed-rank tests to evaluate paired differences between treatment initiation and completion (*α*=0.05). Analysis was performed using Prism 11 (GraphPad, San Diego, CA). Values presented in the text are median (interquartile range), and in the Figures are median (total range).

## 3 Results

### 3.1 Participant characteristics and treatment exposure

Thirty-two men meeting eligibility criteria were included in this case series (Table 2). Median age was 28.9 (24.9–33.0) years, median BMI was 27.7 (24.1–30.7) kg/m^2^, median symptom duration was 18 (9–24) months, and median treatment exposure was 9 (8–12) sessions (Table 2). Symptom duration was 12 months or less in 47% of patients, 13 to 24 months in 31%, 25 to 36 months in 16%, and longer than 36 months in 6%.

**Table 2:** Participant characteristics. Values are median (interquartile range), along with minimum and maximum. *N* = 32 male patients.

Parameter	Median (IQR)	Minimum	Maximum
Age, years	28.9 (24.9–33.0)	20.8	41.2
BMI, kg/m^2^	27.7 (24.1–30.7)	21.7	33.8
Symptom duration, months	18 (9–24)	4	60
Treatment sessions	9 (8–12)	5	15

### 3.2 Clinical presentation and perceived contributing factors

Clinical history features and perceived contributors are shown in Figure 1A. All patients had documented penile morphological changes and reported first identifying the term HFS through internet searches rather than from a clinician. Common sexual features included morning erection alterations (59%), ejaculatory dysfunction (56%), erectile dysfunction (50%), and excessive or aggressive masturbation (41%). Common regional symptoms included low back pain (53%), hip or groin pain (38%), scrotal pain (34%), pelvic floor pain or tightness (31%), urinary symptoms (28%), bowel dysfunction (13%), and abdominal pain or cramping (13%). Psychosocial and lifestyle features included relationship or dating stress related to HFS symptoms (72%), poor sleep (34%), anxiety (31%), problematic pornography use (31%), relationship stress unrelated to symptoms (31%), major life stressor (16%), depression (13%), prolonged sitting (25%), and injury during intercourse (13%).

**Figure 1:**
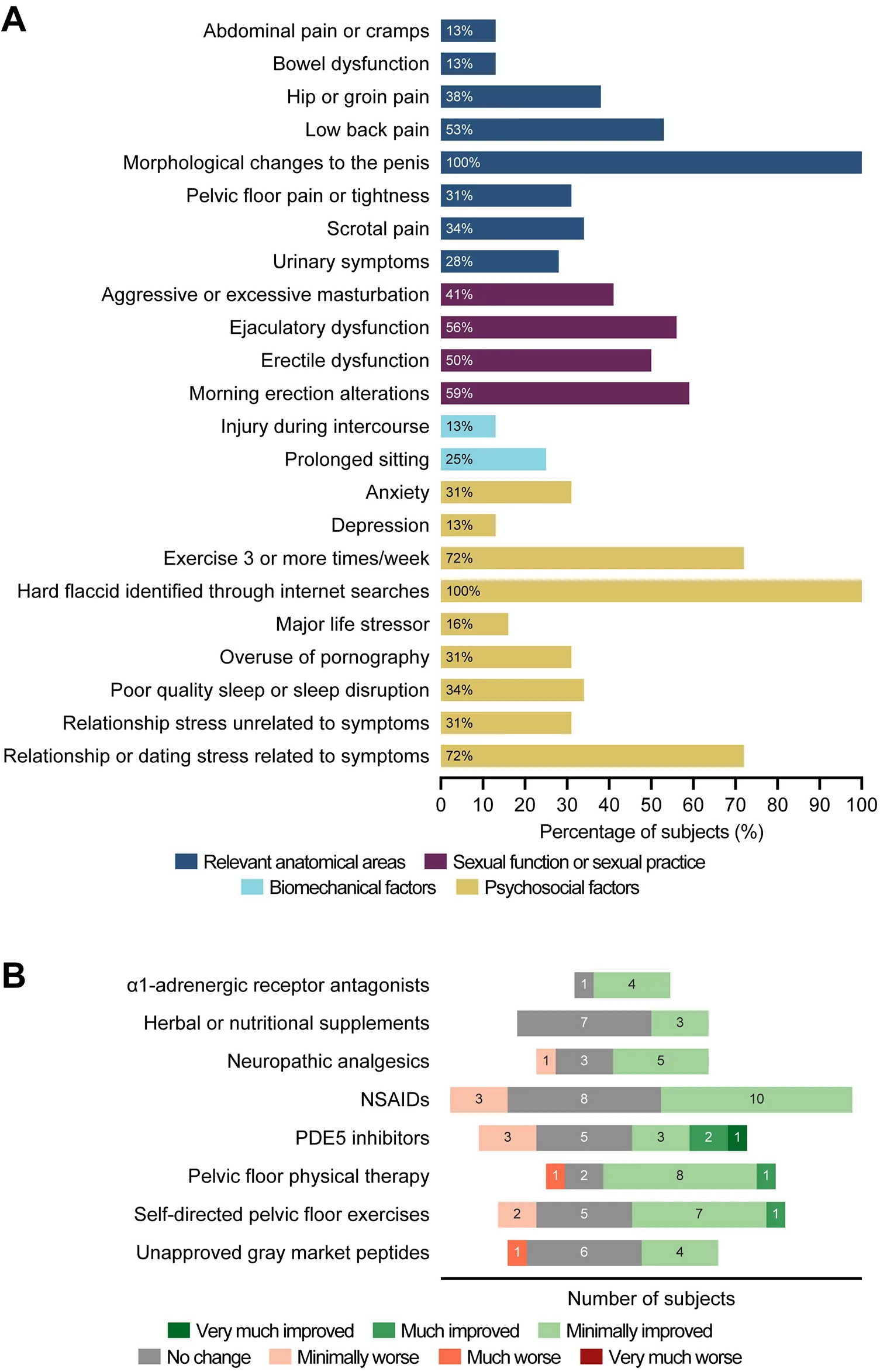
Patient-reported symptom features, perceived contributing factors, and prior treatment responses in patients with hard flaccid syndrome. **(A)** Frequency of documented symptoms, anatomical regions, behavioral factors, sexual function or sexual practice factors, and psychosocial factors identified from the clinical history. Bars represent the percentage of patients with each feature documented in the history of present illness. Categories were not mutually exclusive, and patients could have more than one documented symptom, exposure, or perceived contributor. **(B)** Patient-reported perceived response to previously attempted treatments. Bars represent the absolute number of patients reporting each response category for a given intervention. Response categories ranged from very much worse to very much improved in a 7-point Patient Global Impression of Change scale. PDE5, phosphodiesterase type 5; NSAIDs, nonsteroidal anti-inflammatory drugs.

### 3.3 Prior treatments and patient-reported responses

Prior treatment responses are shown in Figure 1B. NSAIDs were most common (*N* = 21), with 10 patients reporting improvement, 8 no change, and 3 worsening. Self-directed pelvic floor exercises (*N* = 15) and PDE5 inhibitors (*N* = 14) were mixed, with approximately half reporting improvement. Prior pelvic floor physical therapy (*N* = 12) improved symptoms in 9 patients, although all 12 reported discomfort with internal release and preferred to avoid this in future sessions. Unapproved peptides (*N* = 11), neuropathic analgesics (*N* = 9), and herbal or nutritional supplements (*N* = 10) improved symptoms in roughly one-third to one-half of users. One patient experienced worsening after self-injection of the unapproved peptide BPC-157, which is marketed online as a treatment for a variety of conditions despite not having any meaningful human subjects data (56), into his penis outside of medical guidance. Five patients reported *α*1-adrenergic receptor antagonists use, with 4 reporting improvement and 1 reporting no change.

### 3.4 Sexual interest and global health outcomes

PROMIS outcomes improved from treatment start to completion (Figure 2). PROMIS Interest in Sexual Activity increased from 38.4 (32.9–43.9) to 54.8 (49.4–60.3, *P* < 0.01). PROMIS Global Health PCS increased from 50.8 (47.7–54.1) to 54.1 (53.3–57.7, *P* < 0.01), and MCS increased from 50.8 (48.3–53.3) to 53.3 (50.8–56.0, *P* < 0.01). The PCS and MCS changes fell within the 2 to 6 T-score range commonly cited as clinically meaningful, and the sexual interest change exceeded this range (28).

**Figure 2:**
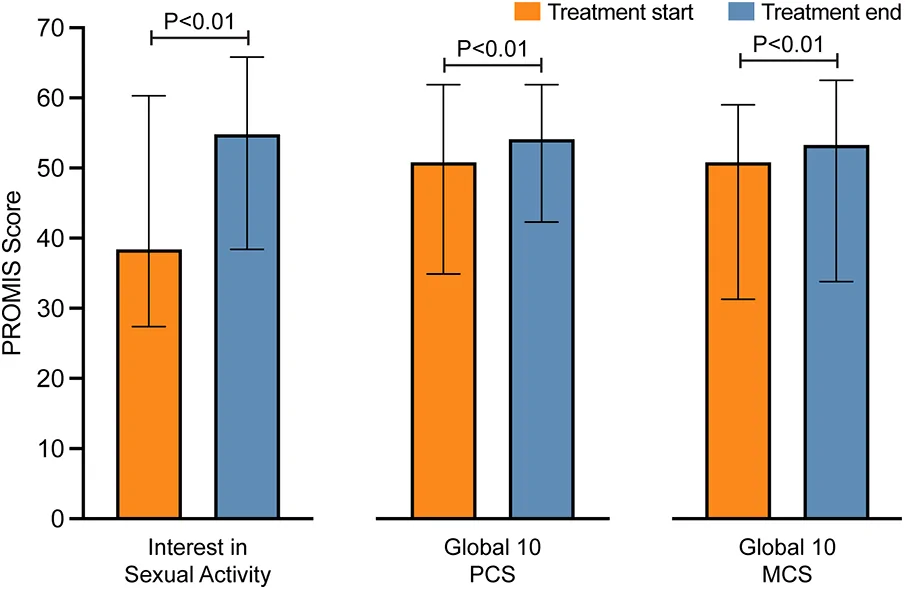
Changes in PROMIS scores from treatment start to treatment end. PROMIS Brief Profile Male Interest in Sexual Activity, PROMIS Global Health-10 physical component score (PCS), and mental component score (MCS). Values are median ± range. Paired comparisons were performed using a two-tailed Wilcoxon matched-pairs signed-rank test. All measures improved from treatment start to treatment end (*P* < 0.01). *N* = 32 patients.

### 3.5 Erection hardness outcomes

EHS improved from treatment start to completion (Figures 3A,B). Median EHS increased from 2 (2–2.25) to 4 (3–4, *P* < 0.01), representing a transition from erections generally insufficient for penetration to erections rated as fully rigid in most patients. At baseline, 7 patients (22%) had EHS 1, 17 (53%) had EHS 2, 6 (19%) had EHS 3, and 2 (6%) had EHS 4. At treatment end, 9 patients (28%) had EHS 3 and 23 (72%) had EHS 4. EHS ≥3 was achieved by 8 of 32 patients (25%) at baseline and 32 of 32 (100%) at treatment end. Overall, 29 patients (91%) improved by at least 1 EHS category, 3 (9%) were unchanged, and none worsened.

**Figure 3:**
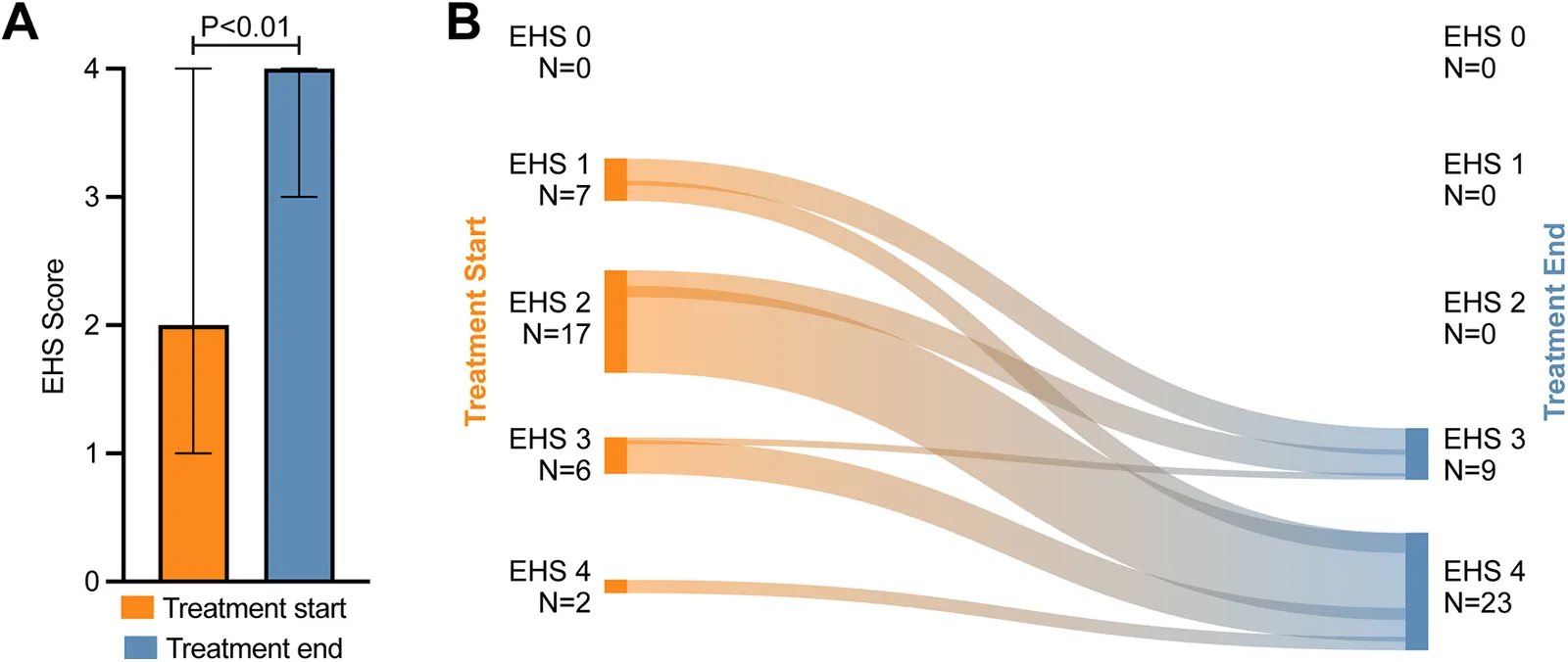
Changes in erection hardness scale (EHS) from treatment start to treatment end. EHS scores at treatment start and treatment end. **(A)** Values are median ± range. Paired comparisons were performed using a two-tailed Wilcoxon matched-pairs signed-rank test. **(B)** Sankey graph demonstrating patient-level transitions in EHS category from treatment start (orange) to treatment end (blue). Flow width represents the number of patients moving from each baseline EHS category to each posttreatment EHS category. Orange nodes indicate treatment start and blue nodes indicate treatment end. EHS: 0, penis does not enlarge; 1, penis is larger but not hard; 2, penis is hard but not hard enough for penetration; 3, penis is hard enough for penetration but not completely hard; 4, penis is completely hard and fully rigid. *N* = 32 patients.

### 3.6 Penile function satisfaction outcomes

Penile satisfaction also improved (Figures 4A,B). Median satisfaction increased from 2 (1, 2) to 5 (5-5, *P* < 0.01). At treatment start, 14 patients were very dissatisfied, 14 were dissatisfied, and 4 were neutral, while none were satisfied or very satisfied. At treatment end, 4 were satisfied and 28 were very satisfied, with no neutral, dissatisfied, or very dissatisfied responses. All 32 patients improved by at least 1 satisfaction category.

**Figure 4:**
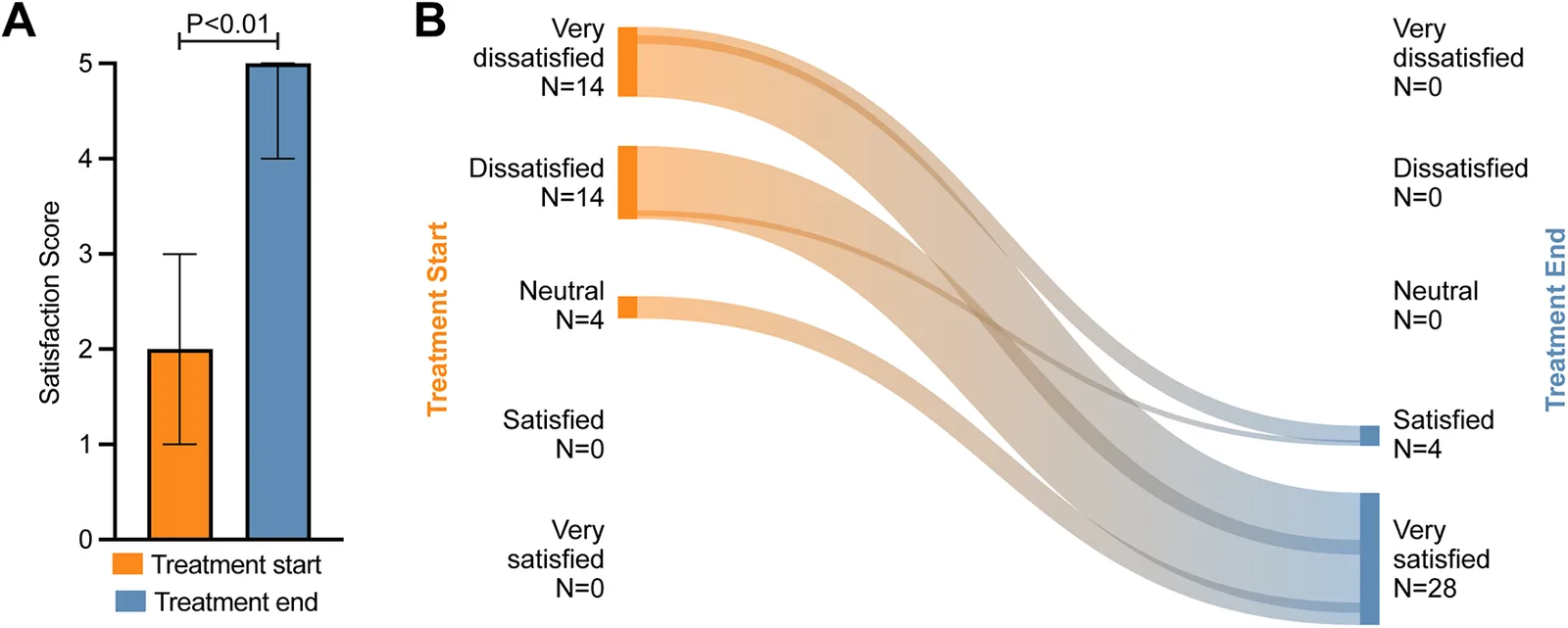
Changes in overall satisfaction with penile function from treatment start to treatment end. **(A)** Values are median ± range. Paired comparisons were performed using a two-tailed Wilcoxon matched-pairs signed-rank test. **(B)** Sankey graph demonstrating patient-level transitions in satisfaction score category from treatment start (orange) to treatment end (blue). Flow width represents the number of patients moving from each baseline satisfaction score category to each posttreatment satisfaction score. Orange nodes indicate treatment start and blue nodes indicate treatment end. *N* = 32 patients.

## 4 Discussion

### 4.1 Principal findings

This retrospective case series of 32 men with HFS is, to our knowledge, the largest treatment-focused report of HFS to date. Limited case studies and small series have indicated success in treating HFS using multimodal treatment programs centered around physical therapy (6–8), leaving uncertainty about whether multimodal treatment would produce consistent outcomes across a broader HFS population. Following a comprehensive multimodal protocol, median EHS improved from 2 to 4, median penile satisfaction improved from 2 to 5, and PROMIS sexual interest, physical health, and mental health scores improved significantly. All patients achieved EHS ≥3 by treatment end, 91% improved by at least 1 EHS category, and satisfaction shifted from universal dissatisfaction or neutrality to universal satisfaction or high satisfaction. These findings support further study of the hypothesis that HFS as a multidimensional condition in which heterogeneous entry points converge on shared neurovascular, sensory, musculoskeletal, and behavioral maintenance pathways. The current study cannot determine treatment efficacy because there was no control group and all patients received multiple interventions, including daily tadalafil. The results instead describe within-patient changes that occurred during a structured multimodal treatment program and provide clinical observations that can be tested in prospective controlled studies.

### 4.2 Hypothesis-generating mechanistic framework

We developed the treatment protocol from available anatomical, physiological, rehabilitation, and sexual medicine evidence, and we present these mechanisms to explain the biological rationale for the intervention and generate testable hypotheses for future studies. At the tissue level, penile rigidity depends on coordinated interactions among cavernosal smooth muscle, superficial fascia, sensory nerves, vascular inflow, and tunical constraint. Cavernosal trabecular smooth muscle maintains flaccidity through tonic *α*1-adrenergic and RhoA/Rho kinase-mediated contraction via sympathetic fibers from T11-L2, while parasympathetic NO/cGMP signaling from S2-S4 promotes relaxation and sinusoidal filling (57, 58). The tunica albuginea provides the passive mechanical constraint against which expanding sinusoids compress the subtunical venular plexus, producing the veno-occlusive mechanism that traps pressurized blood (59). The dartos fascia contains myofibroblasts and smooth muscle under sympathetic *α*1 control, with reflex contributions mapped to L1-L2 via the genitofemoral nerve (60–62). Based on these biological mechanisms, we hypothesize that increased sympathetic outflow could affect cavernosal smooth muscle and dartos fascia through *α*1/RhoA/ROCK signaling and contribute to flaccid semi-rigidity, restricted radial expansion during tumescence, and sensory symptoms observed in patients with HFS.

### 4.3 Hypothesized targets of the multimodal intervention

This framework provides hypotheses for how individual components of the protocol could act through partially overlapping pathways (Figures 5, 6). First, studies outside HFS indicate that dry needling can create a focal strain field in connective tissue and induce cytoskeletal remodeling and myofibroblast relaxation (63). Needle stimulation can release adenosine from myofibroblasts, which may inhibit nociceptive afferent transmission and reduce sympathetic drive (64). Needle stimulation may also promote local NO production and reduce RhoA/ROCK-mediated cell contraction (64). Second, preclinical studies indicate that shockwave therapy can reduce myofibroblast tension through RhoA/ROCK signaling and may reduce *α*1-adrenergic receptor expression (65), which could decrease tonic activation of both dartos fascia and cavernosal tissue. Third, photobiomodulation may modify RhoA/ROCK activity through cGMP signaling (66), and in the long-term by reducing smooth muscle actin expression (67). Photobiomodulation may also modulate TRPV channels in nociceptive neurons (68). Fourth, experimental studies indicate that thermal ultrasound can activate NO signaling, inhibit ROCK activation, and alter myofibroblast tension (69, 70). Fifth, transverse loading of connective tissue may alter tension anisotropy and reduce myofibroblast contractile force (71). Finally, tadalafil sustains cGMP levels by inhibiting PDE5, which deactivates the RhoA/ROCK calcium-sensitization pathway that maintains tonic contraction in smooth muscle cells (72). Although not part of our case series treatment protocol, *α*1-adrenergic receptor antagonists directly target the activator arm of the same pathway, and 4 of 5 patients in our cohort who had previously used *α*1-AR antagonists reported symptomatic improvement. While the current study did not measure these pathways and cannot determine whether any component acted through the proposed mechanisms, we hope that additional prospective and mechanistic studies will provide greater insight into the tissue-level pathology of HFS.

**Figure 5:**
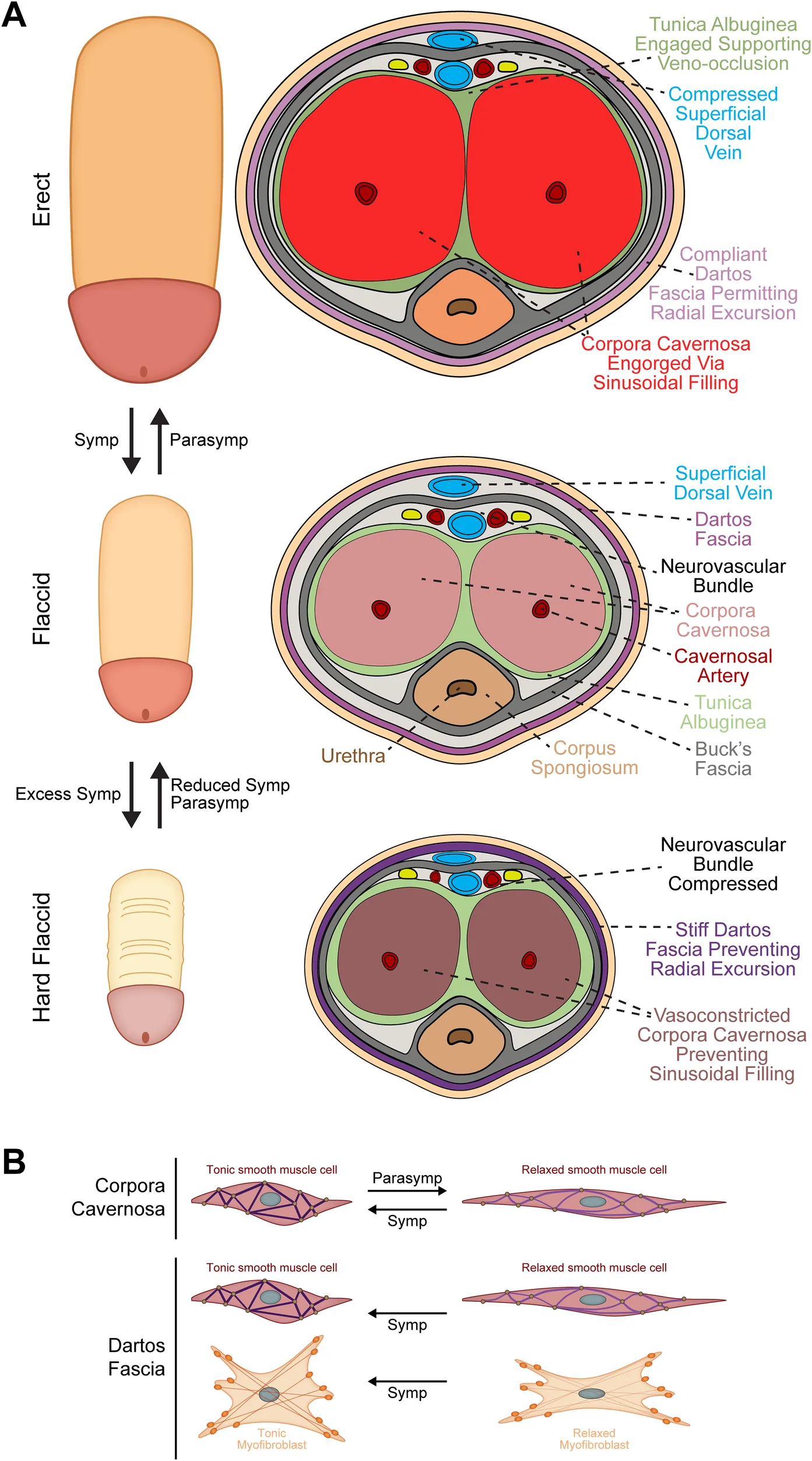
Proposed cellular and tissue-level mechanisms in hard flaccid syndrome. **(A)** Schematic of the penis in the erect, flaccid, and hard flaccid states, under sympathetic (Symp) and parasympathetic (Parasymp) control. **(B)** Cavernosal smooth muscle cells transition between contraction and relaxation under reciprocal sympathetic and parasympathetic control, while dartos smooth muscle cells and myofibroblasts are regulated by sympathetic input alone.

**Figure 6:**
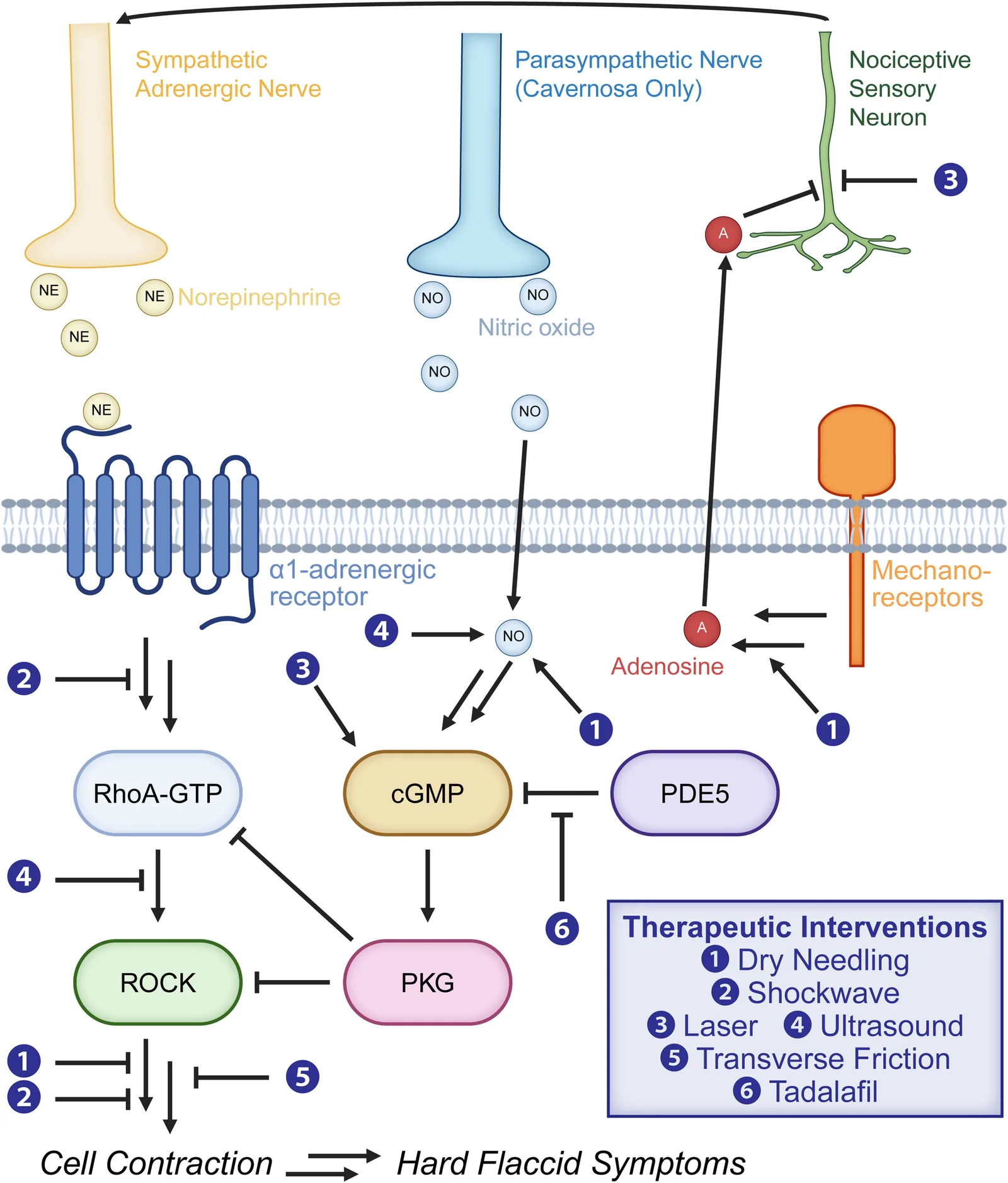
Proposed cellular targets of therapeutic interventions for hard flaccid syndrome. Signaling pathways governing penile rigidity andthe sites of action of therapeutic interventions: (1) dry needling, (2) shockwave, (3) class IV laser, (4) therapeutic ultrasound, (5) transverse friction, and (6) tadalafil.

### 4.4 Integrative model of hard flaccid syndrome

Building on the model proposed by Goldstein and colleagues (5), we hypothesize that musculoskeletal, fascial, or end organ urogenital pathology may contribute to HFS, while central and peripheral nervous system processes may modify or sustain symptoms (Figure 6). Musculoskeletal and fascial contributors include lumbosacral and hip musculoskeletal pathology, primary pelvic floor dysfunction, mechanical nerve injuries, and myofascial dysfunction. End organ contributors include penile and perineal trauma, cavernosal dysfunction, and dartos hypercontraction. In men that are approximately 20 to 40 years of age, low back pain is reported in approximately 12%, hip pain in approximately 5% to 10%, and pelvic floor symptoms in approximately 1.8% (73–75). Each of these was several-fold more prevalent in this cohort. Position-dependent symptoms in HFS, with improvement when lying down and worsening when standing, are consistent with potential spinal and pelvic mechanisms (2). A potential hip contribution is consistent with reports that femoroacetabular impingement can impair sexual function, with over two-thirds of FAI patients reporting sexual difficulties (76). The genitofemoral, ilioinguinal, and iliohypogastric nerves arise from T12-L2, the same spinal levels as sympathetic preganglionic outflow to the hypogastric plexus, while the pudendal and sciatic nerves share S2-S3 roots (77). Sustained nociceptive afferent traffic from back or hip pain generators could converge on dorsal horn neurons and increase segmental sympathetic outflow to cavernosal and dartos tissues through the hypogastric plexus (78, 79). Although the current study did not directly test this pathway, these anatomical relationships provide a plausible route through which peripheral musculoskeletal and end organ pathology could influence a shared autonomic and effector system.

Penile and perineal trauma have also been cited as initiating mechanisms in HFS (4). In this cohort, 41% reported aggressive or excessive masturbation and 13% reported injury during intercourse as perceived contributing factors. Rather than requiring frank structural damage, cumulative mechanical loading could increase nociceptive afferent traffic, promote protective pelvic floor guarding, and reinforce sympathetic outflow to cavernosal and dartos tissues (80–83). Reward circuit activation during sexual behavior may reinforce repeated exposure to sexual activities involving penile loading, while also blunting nociceptive feedback that would otherwise limit continued loading of an injured tissue (84). Combined, we hypothesize that these factors could generate a self-perpetuating loop of tissue damage and sympathetic outflow that persists long after each individual loading session, resulting in an overuse injury occurring through accumulated exposure enhanced by reward-circuit sensitization.

### 4.5 Relationship to contemporary penile rehabilitation

The current treatment protocol for HFS shares several features with contemporary rehabilitation for other penile conditions. Penile rehabilitation after prostatectomy commonly combines PDE5 inhibition with physical or restorative interventions to support erectile function and preserve cavernosal tissue during recovery from cavernous nerve injury. These approaches can include PDE5 inhibitors, vacuum erectile devices, pelvic floor muscle training, and low-intensity extracorporeal shockwave therapy (11). Although there is overlap between these approaches and our HFS protocol, the proposed treatment targets are different. Post-prostatectomy penile rehabilitation primarily seeks to reduce the effects of cavernous nerve injury, penile hypoxia, smooth muscle loss, fibrosis, and veno-occlusive dysfunction (11). In contrast, HFS may involve pelvic floor overactivity, altered penile sensation, regional musculoskeletal pathology, fascial dysfunction, autonomic dysregulation, and behavioral or psychosocial contributors (5, 51). In the current study, tadalafil and penile-directed treatments therefore overlap with conventional penile rehabilitation, while pelvic floor rehabilitation, musculoskeletal treatment, neuromodulation, therapeutic exercise, and behavioral interventions address additional features of HFS.

### 4.6 Limitations

Several limitations should be considered. Because care was delivered in routine clinical practice, the primary goal was to optimize patient outcomes rather than isolate treatment effects under randomized conditions. The protocol therefore combined the best available modalities, as is common in clinical rehabilitation, but this approach prevents determination of the independent contribution of each intervention. The retrospective uncontrolled design without a comparator group precludes attribution of improvement to any specific intervention, and the multimodal nature of the protocol prevents component-wise efficacy estimation. The single-center setting may limit generalizability to other clinical contexts, and the assessment window did not include long-term follow-up beyond treatment completion. The absence of validated HFS-specific patient-reported outcome instruments required adaptation of erectile function and satisfaction measures developed for other populations, and self-reported outcomes are vulnerable to expectation effects. We did not routinely collect the International Index of Erectile Function-5 or -15 because several items depend on recent partnered intercourse, which limited applicability in patients who were single or avoiding intercourse because of HFS. We therefore used the EHS to assess erection hardness independent of recent intercourse. We required persistent or recurrent semi-rigidity of the flaccid penis for study inclusion, but we did not quantify the severity or frequency of this finding longitudinally. Penile duplex Doppler ultrasound was not performed because several patients reported symptom worsening after prior testing. No validated HFS-specific patient-reported outcome measure exists, requiring use of validated general instruments and an unvalidated penile satisfaction item. Finally, the proposed model is hypothesis-generating and extrapolates from adjacent anatomical, physiological, and treatment-response literature. The proposed model therefore remains a hypothesis and requires prospective testing with controlled designs, objective measures, and predefined treatment protocols.

### 4.7 Conclusions

Goldstein and colleagues (5) positioned HFS within the broader spectrum of (PGAD/GPD) syndromes driven by the pelvic-pudendal-hypogastric reflex. Our findings support a refinement in which musculoskeletal, fascial, and end organ contributors may converge through autonomic, somatosensory, and effector pathways, while central and peripheral nervous system processes amplify, modify, or sustain symptoms (Figure 7). In this case series, multimodal rehabilitation was associated with substantial improvements in erectile hardness, sexual interest, penile satisfaction, and global physical and mental health. These findings support diagnostic screening for musculoskeletal, fascial, end organ, sensory, autonomic, and behavioral contributors and provide a rationale for prospective controlled trials. Future work that formally characterizes the relative contribution of each component in HFS populations, develops validated HFS-specific patient-reported outcome instruments, and prospectively tests the specific predictions of the model in a controlled trial would advance the treatment of HFS and other penile and pelvic disorders.

**Figure 7:**
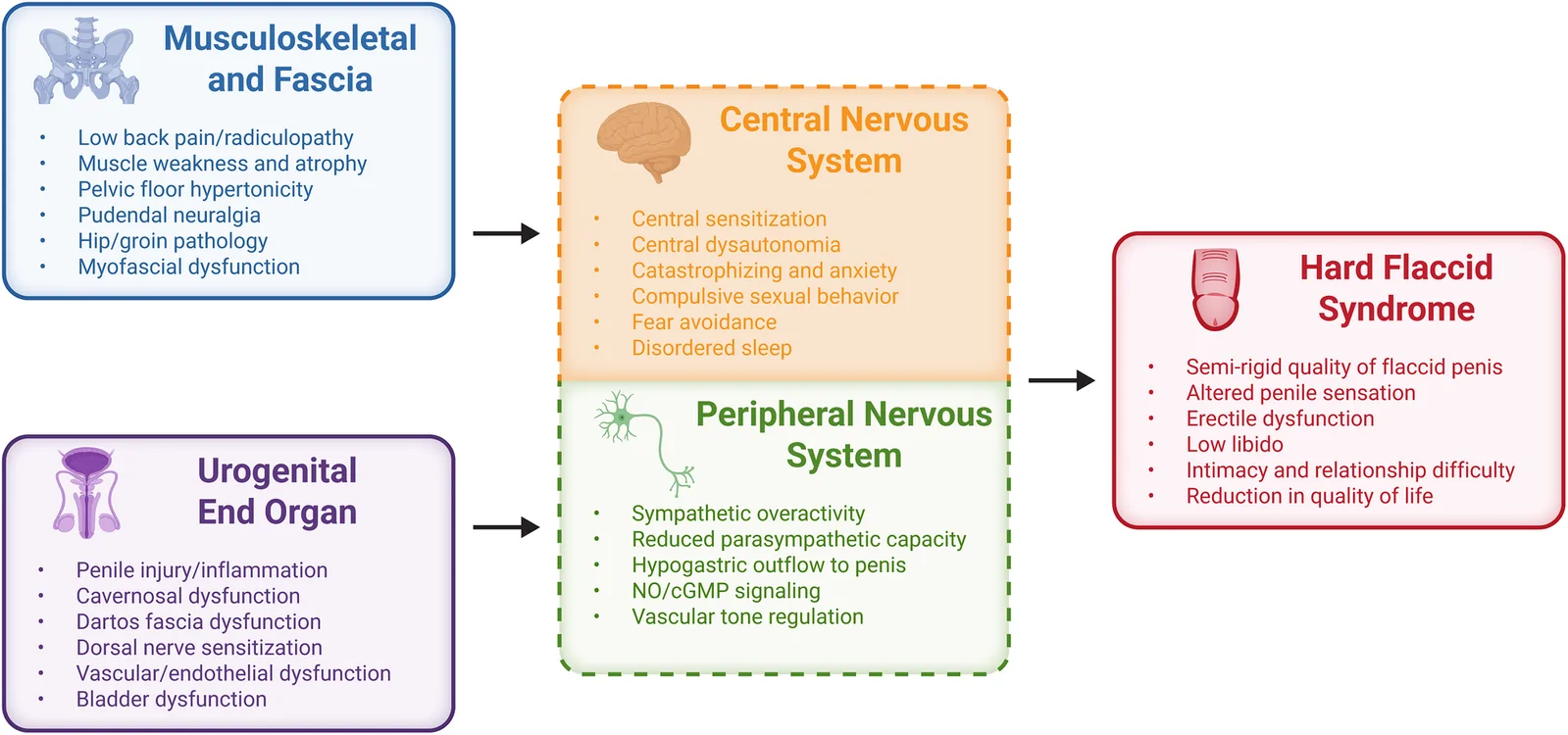
Proposed integrative model of hard flaccid syndrome. Musculoskeletal and fascia pathology and end organ urogenital pathology contribute to hard flaccid syndrome through integration by the central and peripheral nervous systems. The resulting clinical phenotype includes the persistent semi-rigid quality of the flaccid penis along with broader features of sexual dysfunction, intimacy difficulty, and reduced quality of life.

## Data Availability

De-identified data will be made available upon reasonable request to the corresponding author.
